# Antagonism of β-klotho signaling by peptide 19 impairs wheel running in male mice and potentiates the cisplatin-induced decrease in wheel running

**DOI:** 10.3389/fphar.2026.1800474

**Published:** 2026-06-26

**Authors:** Brandon M. Chelette, Lisa Abe, Chinenye Chidomere, Richard D. DiMarchi, Fa Zhang, Robert Dantzer

**Affiliations:** 1 Department of Symptom Research, The University of Texas MD Anderson Cancer Center, Houston, TX, United States; 2 Baylor College of Medicine, Houston, TX, United States; 3 Department of Chemistry, Indiana University Bloomington, Bloomington, IN, United States

**Keywords:** Beta-Klotho, cancer-related fatigue, energy metabolism, FGF21, mouse

## Abstract

**Background:**

Cancer-related fatigue results from competition between the metabolic demands of the tumor and those of skeletal muscles. This competition leads to an energy metabolism deficit, which is worsened by the detrimental effects of cancer therapy on mitochondrial function. Mitokines, such as Fibroblast Growth Factor 21 (FGF21), produced under these conditions help tissues adjust their metabolism to cope with cellular stress. However, the metabolic changes they trigger can be harmful if these alterations persist and become uncontrolled. Although FGF21 is involved in the metabolic adaptations needed for physical exercise, its potential role in cancer-related fatigue has not yet been studied. FGF21 has low affinity for FGF Receptors (FGFRs) and requires the co-receptor Beta-Klotho (KLB) to act in tissues such as the liver, adipose tissue, and brain.

**Methods:**

To determine whether FGF21 is produced in response to cancer and its therapy, FGF21 levels were measured in the plasma using ELISA and in the liver by quantitative real time polymerase chain reaction (qRT-PCR) in male mice administered a human papilloma virus-related head and neck cancer tumor cell line or treated with the chemotherapeutic agent cisplatin. To examine the role of FGF21 in cancer-related fatigue, tumor-bearing male mice or cisplatin-treated male mice trained to run on a wheel were given an analog of FGF21 (LY2405319) and a β-klotho antagonist (peptide-19). Behavioral fatigue was assessed by the reduction in voluntary wheel running caused by these interventions.

**Results:**

Both tumor growth and cisplatin increased circulating FGF21 levels and liver mRNA expression of FGF21. Chronic administration of LY2405319 at two doses did not affect wheel running in healthy mice. In contrast, administration of peptide 19 worsened behavioral fatigue caused by tumor progression and had a similar negative effect on behavioral fatigue in cisplatin-treated mice.

**Conclusion:**

By demonstrating that blocking activation of β-klotho, a co-receptor for FGF21 and other fibroblast growth factors, exacerbates cancer-related fatigue, the present findings underscore the significance of the production of these endocrine signals in enabling mice to maintain physical activity when their energy metabolism is compromised by cisplatin chemotherapy.

## 1 Introduction

The mitochondrial unfolded protein response develops in response to stressors such as excessive oxidative stress and mitochondrial DNA damage. It leads to the production and release of signaling factors known as mitokines. Mitokines act locally, in an autocrine and paracrine manner, to help cells respond adequately to the stressor, and at a distance, in an endocrine manner, to regulate the necessary metabolic adjustments at the organismal level (Burtscher et al., 2023; Zhang et al., 2024). Although this helps maintain energy homeostasis in the short term, a sustained increase in circulating mitokine levels can cause metabolic disorders. This is the case, for instance, with one of the main mitokines, Growth Differentiation Factor 15 (GDF15). By activating its brain-specific receptor, the Glial Cell-Derived Neurotrophic Factor Family Receptor Alpha-Like (GFRAL), GDF15 induces anorexia and increases energy expenditure, resulting in substantial body mass loss and ultimately leading to cachexia in cancer patients (Emmerson et al., 2017; Kim-Muller et al., 2023; Groarke et al., 2024). These effects are preceded by a reduction in physical performance, which could be secondary to alterations in the mechanisms that regulate the provision and utilization of energy substrates by skeletal muscles, adipose tissue, and the liver during physical exercise. Another mitokine of potential importance in this regard is Fibroblast Growth Factor 21 (FGF21). Its regulatory effects on lipid and glucose metabolism are mediated at the level of metabolically active organs and the brain by the co-expression of its receptor, FGFR, and its obligatory coreceptor, β-klotho (KLB) (Tezze et al., 2019; Ogawa et al., 2007; Sullivan et al., 2024). Like GDF15, FGF21 is produced by several cancer types, where it can promote or inhibit tumor progression by modulating cellular proliferation or tumor immunity (Qian et al., 2018; Dai et al., 2021; Hu et al., 2024).

Fatigue is a common symptom in cancer patients. It is often present at diagnosis, increases notably during treatment, and does not consistently improve after therapy ends (Dantzer et al., 2025). At the behavioral level, fatigue is usually characterized by an inability to sustain physical activity. We previously reported that behavioral fatigue in tumor-bearing mice results from competition between the energy demands of the tumor and those of skeletal muscles, exacerbated by chemotherapy-induced mitochondrial damage (Dantzer et al., 2025). GDF15, which increases dramatically in circulation in response to chemotherapy, appears to be the primary cause of behavioral fatigue, as neutralizing GDF15 reduces its development and aids recovery (Chelette et al., 2023). Although FGF21 is known to play a role in metabolic adaptations during physical exercise (Abe and Dantzer, 2025), its role in cancer-related fatigue has not been investigated. The present study, conducted in mice, aims to fill this knowledge gap. We demonstrate that cisplatin significantly elevates circulating FGF21 levels, and this effect is also observed in tumor-bearing mice. Additionally, administering an FGF21 agonist does not affect voluntary wheel running, while blocking its coreceptor with peptide 19 worsens behavioral fatigue caused by cisplatin and the tumor. These results suggest that the metabolic effects of FGF21 likely help maintain a basic level of physical functioning during cancer and its treatment.

## 2 Animals and methods

### 2.1 Animal care and ethical statement

All procedures received approval from the Institutional Animal Care and Use Committee (IACUC) of the University of Texas MD Anderson Cancer Center (UT MDACC; Houston, TX, United Ststes) under protocol #00002034-RN01 and were conducted in accordance with the guidelines in the NIH Guide for the Care and Use of Laboratory Animals.

### 2.2 Animals

The experiments were conducted exclusively in male mice, as the tumor cell line under study does not grow in female mice (see Section 2.4, Tumor model, below). C57BL/6 J mice, aged 10–12 weeks, were obtained from The Jackson Laboratory (Bar Harbor, ME, United States) and housed in the animal facility at the University of Texas MD Anderson Cancer Center. After a 1-week quarantine and acclimation period following their arrival, the mice were housed individually with free access to food (5053 Irradiated Rodent Diet 20; 24.5% kcal from protein, 13.1% fat, 62.4% carbohydrates; LabDiet, St Louis, MO, United States) and water. They were maintained on a 12:12-h light/dark cycle, with lights off at 19:00 CST and on at 07:00 CST. For experiments measuring behavioral fatigue through decreases in wheel running, mice were provided with a Low-Profile Wireless Running Wheel (Med Associates Inc., St. Albans, VT, United States) in their home cages. Every 30 s, each wheel transmits a data packet to a central hub that records the number of revolutions. This data is stored by the Wheel Manager software (Med Associates Inc.) and exported for further analysis. Since most running occurs during the dark phase of the cycle, voluntary wheel-running activity was measured by the number of revolutions during the night.

### 2.3 Drug preparation and administration

Cisplatin (Accord Healthcare Inc.; Durham, NC, United States) was diluted in sterile PBS and administered intraperitoneal at 10 mg/kg for acute treatment. For chronic treatment, cisplatin was administered daily intraperitoneal at a dose of 2.83 mg/kg for one 5-day cycle. These doses are commonly used by our lab to induce mitochondrial dysfunction and the integrated stress response (Chelette et al., 2023).

LY2405319, a FGF21 variant designed to improve protein expression and formulation stability (Kharitonenkov et al., 2013), was diluted in sterile PBS and administered subcutaneously daily at a dose of 3 or 7.5 mg/kg, which improved metabolic dysfunction in male *ob/ob* mice (Kharitonenkov et al., 2013) and in a mouse model of type I diabetes (Kim et al., 2015). For both cisplatin and LY2405319, control mice received an injection of sterile Phosphate-Buffered Saline (PBS), replicating the timing, volume, and route of the active compound injections.

Peptide 19 was designed as a fatty-acetylated peptide to prolong the half-life and enhance the biological activity of peptides that serve as specific antagonists of the KLB co-receptor (Pan et al., 2020). When administered daily at a dose of 3 mg/kg, it completely inhibited the metabolic effects of FGF19 and FGF21 in a diet-induced mouse model of obesity (Pan et al., 2020). To block the biological activity of endogenous FGF21 produced in response to cisplatin and cancer, mice were given the same dose of peptide 19 daily via subcutaneous injection. Control mice received an equivalent volume of sterile PBS.

### 2.4 Tumor model

To determine whether FGF21 plays a role in tumor-induced fatigue, we selected a highly metabolically active murine model of human papilloma virus (HPV)-related head and neck cancer consisting of male epithelial oropharyngeal cells transfected with HPV type 16 oncogenes E6 and E7 and H-RAS (mEER tumor cell line) (Grossberg et al., 2020; Spanos et al., 2009; Coppock et al., 2013). Mice were injected with 1 × 10^6^ mEER cells in 50 μL of sterile PBS into the flank. Control mice received an equivalent volume of PBS. Tumor weight was measured at the end of the experiment.

### 2.5 Experimental design

#### 2.5.1 Experiment 1: effects of cisplatin on levels of FGF21 in the plasma and on the mRNA expression of *Fgf21* in the liver

To determine whether cisplatin induces FGF21, mice were injected with 10 mg/kg cisplatin or PBS (n = 6/group). Blood was collected 6 h later via cardiac puncture in anesthetized mice exposed to CO_2_. Blood samples were held on ice until centrifugation at 4,000 × g at 4 °C. The plasma was then collected and stored at −80 °C until analysis. FGF21 concentration in the plasma samples was determined via ELISA (Mouse FGF21 DuoSet ELISA; Cat #DY3057; R&D Systems, Inc., Minneapolis, MN, United States). Following blood collection, mice were perfused with 20 mL of ice-cold PBS and the liver was excised and snap frozen in liquid nitrogen. Liver samples were stored at −80 °C until mechanical homogenization via liquid nitrogen-chilled mortar and pestle followed by RNA extraction with RNA-Solv according to the manufacturer’s instructions (Omega Bio-Tek, Norcross, GA, United States). RNA concentration was determined via NanoDrop One microvolume spectrophotometer (Thermo Fisher Scientific). All RNA samples were then diluted to the same concentration (100 ng/μL) and reverse transcribed to cDNA using the High-Capacity cDNA Reverse Transcription Kit according to the manufacturer’s instructions (Thermo Fisher Scientific; Cat #43-688-14). Relative expression (calculated with the 2^−ΔΔCt^ method normalized to the PBS-treated samples) of *Fgf21* was determined via RT-qPCR using iTaq Universal SYBR Green Super Mix (Bio-Rad Laboratories Inc.) and pre-designed oligo primers targeting *Fgf21* (Integrated DNA Technologies, Assay ID: Mm.PT.58.29365871.g) according to the manufacturer’s instructions.

#### 2.5.2 Experiment 2: effects of cancer on plasma levels of FGF21 and the mRNA expression of *Fgf21* in the tumor and liver

To determine whether mEER tumor cells induce FGF21, mice were implanted with 1 × 10^^6^ tumor cells in their flanks. Control mice received PBS in the same anatomical localization. The sample size consisted of 8 mice per group. At the end of the experiment, mice were anesthetized by CO_2_ exposure 21 days later to obtain plasma samples as in Experiment 1. FGF21 levels were measured by ELISA in plasma samples. Liver samples and tumor samples were collected and processed in the same manner as Experiment 1. Tumor RNA samples were diluted to 50 ng/μL, but all other steps remained the same. Relative expression of *Fgf21* was calculated using the 2^−ΔΔCt^ method, normalized to the non-tumor implanted mouse liver samples.

#### 2.5.3 Experiment 3: effects of LY2405319 on voluntary wheel running

To determine whether exogenous FGF21 affects voluntary wheel-running activity, mice trained to run on a wheel for 2 weeks were injected subcutaneously with either LY2405319 (3 mg/kg daily) or the vehicle for 5 days. After a 1-week washout period, the LY2405319 dose was increased to 7.5 mg/kg daily for 4 days. Each administration of LY2405319 was followed by a subcutaneous injection of peptide 19 (3 mg/kg) or its vehicle. This was conducted in a 2 (± peptide 19) x 2 (±LY2405319) factorial design with n = 6 mice per group. Wheel-running activity was monitored during treatment and up to 3 or 7 days afterward.

#### 2.5.4 Experiment 4: effects of peptide 19 on cisplatin-induced decrease in voluntary wheel running

To determine whether endogenous FGF21 contributes to the decrease in voluntary wheel running caused by cisplatin, mice were pretreated daily with peptide 19 (3 mg/kg, subcutaneous injection) or its vehicle, followed by daily injections of cisplatin (2.83 mg/kg, intraperitoneal injection) or its vehicle for 5 days. This followed a 2 (± peptide 19) x 2 (±cisplatin) factorial design, with n = 6 mice per group. Wheel-running activity was recorded before, during, and up to 10 days after treatment. Because cisplatin causes body weight loss, body weight was measured daily during this period.

#### 2.5.5 Experiment 5: effects of peptide 19 on tumor-induced decrease in voluntary wheel running

To determine whether endogenous FGF21 plays a role in the decrease in voluntary wheel running induced by cancer, mice were injected subcutaneously with 1 × 10^6^ mEER tumor cells in their flank or an equivalent volume of PBS (Day 0). When their wheel-running activity began to decline 5 days later (Day 4), mice were injected subcutaneously with peptide 19 (3 mg/kg/day) or its vehicle for 14 consecutive days. Wheel running activity was recorded before, during, and 1 day after treatment.

### 2.6 Statistical analysis

Concentrations of FGF21 in the plasma samples of PBS-injected and cisplatin-injected mice were compared using an unpaired Student’s t-test. The same statistical analysis was used to compare plasma levels of FGF21 in tumor-bearing and control mice. Body weights and wheel running were expressed as a percentage of the baseline measured during the last 3 days before the intervention. The corresponding values were analyzed with a 3-way ANOVA, with the experimental day as a repeated measure and ± LY2405319 and ± peptide 19 as between-subject factors for the experiment on the effects of FGF21 on wheel running. The same statistics were used to analyze the impact of peptide 19 on cisplatin-induced behavioral fatigue and body weight, both expressed as a percentage of baseline values. The between-subject factors were ± cisplatin and ± peptide 19. Post hoc group comparisons were performed at each time point using the Tukey method to correct for multiple comparisons. The impact of peptide 19 on wheel running performance of tumor-bearing mice, expressed as a percentage of baseline, was assessed using three experimental groups (no tumor + PBS; tumor + PBS; and tumor + peptide 19). The corresponding data were analyzed using a 2-way ANOVA with time as a repeated factor. The significance threshold for all analyses was set at α = 0.05. Data in graphs are presented as group means ± standard error of the mean (SEM). Data were organized and stored in Microsoft Excel. Statistical tests were conducted, and graphs were generated using GraphPad Prism (Version 9.0.0, GraphPad Software, San Diego, CA, United States). Graphical figures were designed using the open-source software Inkscape (Inkscape Project, inkscape.org). The detailed results of the 3-way and 2-way ANOVAs are provided in the Supplementary Section.

## 3 Results

### 3.1 Cisplatin and HPV-related head and neck tumors increase plasma levels of FGF21 and the expression of *Fgf21* mRNA in the liver

**FIGURE 1:**
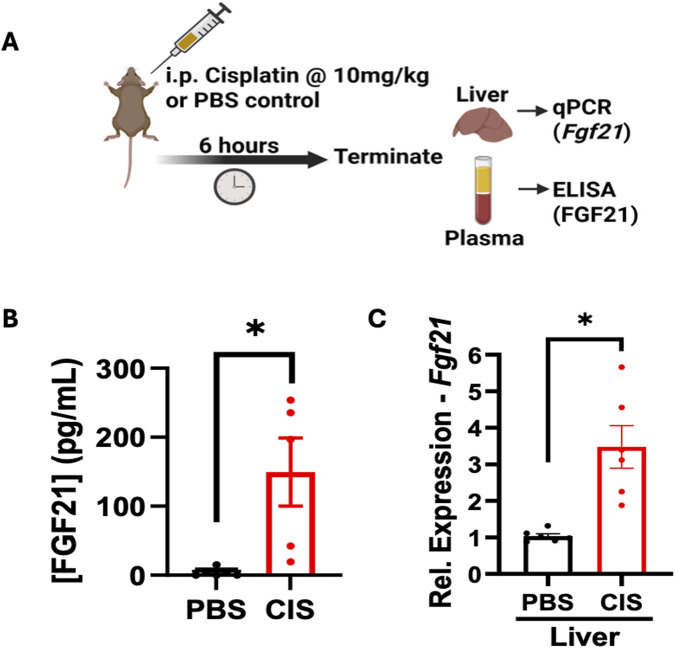
Chemotherapy model of mitokine induction. A schematic of the experimental design **(A)** and the resulting quantification of circulating FGF21 via ELISA **(B)** and relative expression of *Fgf21* mRNA in the liver via qPCR **(C)**. Sample sizes as follows: ELISA–cisplatin (n = 5) and PBS (n = 6) | qPCR–cisplatin (n = 6) and PBS (n = 6). * indicates statistical significance according to unpaired Students t-test. i.p. = intraperitoneal, cis = cisplatin. Bars indicate group means. Error bars indicate standard error of the mean. Symbols represent individual mice.

**FIGURE 2:**
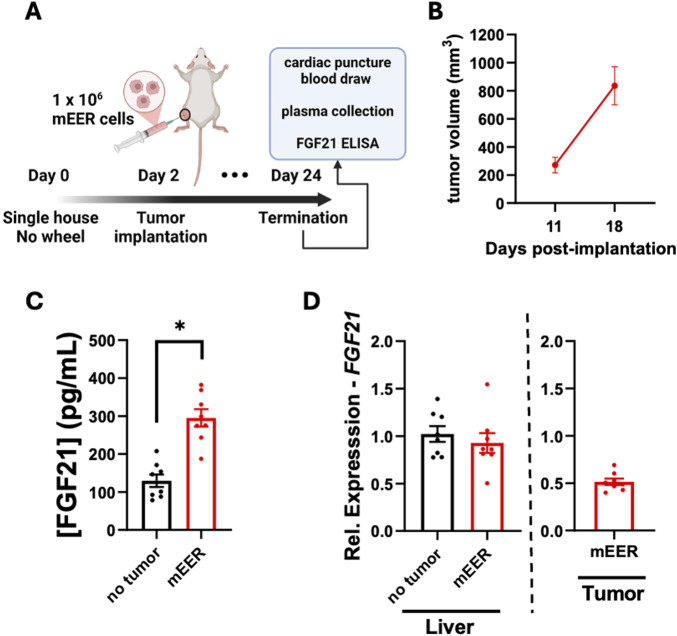
Tumor model of mitokine induction. A schematic of the experimental design outlining the timeline of tumor implantation **(A)**. Tumor volumes estimated via caliper measurements in three orthogonal directions and the formula: (π/6)*(length x width x height) **(B)**. The plasma levels of FGF21 quantified via ELISA **(C)**. RT-qPCR results for *Fgf21* mRNA expression in the liver and tumor **(D)**. Sample sizes as follows: mEER-tumor-bearing mice (n = 8) and non-tumor controls (n = 8). * indicates statistical significance according to unpaired Student’s t-test. Bars indicate group means in **(C,D)**. Error bars indicate standard error of the mean. Symbols represent individual mice in **(C,D)**, and group means in **(B)**.

To determine whether cancer and its treatment affect the production and release of FGF21, mice were either treated with cisplatin or implanted with mEER tumor cells. In both cases, the intervention resulted in an increase in plasma levels of FGF21 (t(9) = 3.3, p < 0.01 for cisplatin; Figure 1) and (t(14) = 5.9, p < 0.001; Figure 2 for mEER tumor) and in the case of the liver, for the expression of *Fgf21* mRNA in response to cisplatin (t(10) = 4.2, p < 0.01). The mEER tumor expressed *Fgf21* mRNA but its progression was not associated with increased expression of *Fgf21* mRNA in the liver (Figure 2).

### 3.2 LY2405319 has no effect on voluntary wheel running activity in healthy mice

**FIGURE 3:**
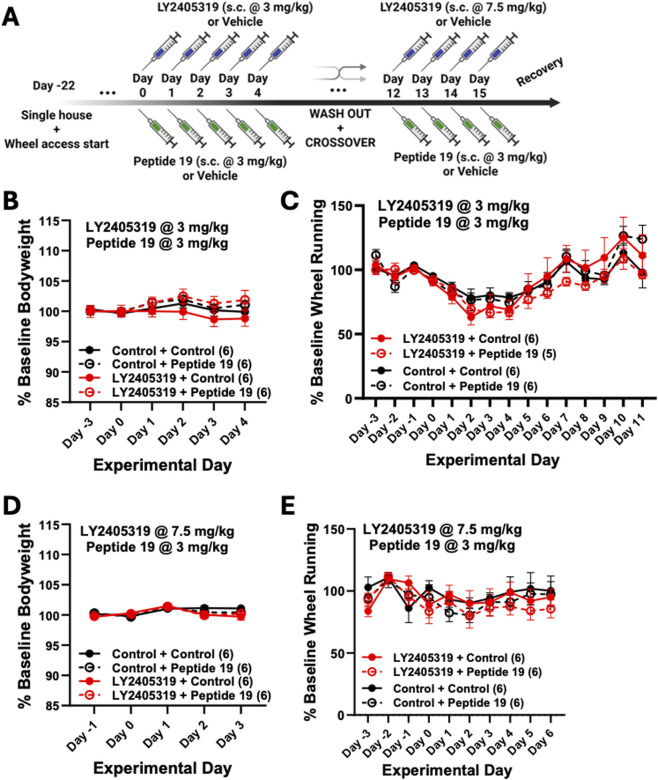
Effects of LY2405319 and peptide 19 co-administration on body weight and wheel running. A schematic of the experimental design outlining the timeline of LY2405319 and Peptide 19 administration **(A)**. The percent bodyweight of both the low (3 mg/kg) and high (7.5 mg/kg) doses of LY2405319 with and without Peptide 19 tracked over the experimental duration **(B,D)**. Wheel running activity presented as percent of a 3-day pretreatment baseline of both the low and high doses of LY2405319 with and without Peptide 19 tracked over the experimental duration **(C,E)**. Symbols indicate group means. Error bars indicate the standard error of the mean.

Mice ran an average of 34,593 ± 1,997 revolutions (mean ± SEM) per night at baseline. To determine whether exogenous FGF21 modifies wheel running, mice were administered daily doses of LY2405319 preceded or not by injection of the KLB antagonist, peptide 19, to assess the specificity of the effect of the FGF21 agonist. Wheel running activity expressed as a percentage of baseline activity was not affected by the repeated administration of LY2405319 at 3 mg/kg (F(1,19) = 0.74) and the specific KLB antagonist, peptide 19 (F(1,19) = 0.25) (Figure 3). The same negative result was observed for body weight expressed as a percent of baseline (F(1,20) = 0.10 for LY2405319 and F(1, 20) = 2.82 for peptide 19). Increasing the daily dose of LY2405319 from 3 to 7.5 mg/kg did not alter this negative result (F(1, 20) = 0.05 for wheel running, F(1, 20) = 2.10 for body weight).

### 3.3 Peptide 19 potentiates the decrement in wheel running induced by cisplatin, but has no effect on the cisplatin-induced decrease in body weight

**FIGURE 4:**
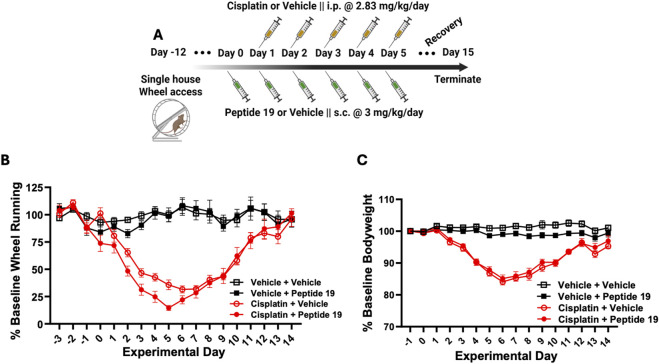
Peptide 19 in cisplatin-treated mice. **(A)** Experimental timeline and design. **(B)** Line graph showing the change in percent baseline wheel running over time **(C)** Line graph showing the change in percent baseline bodyweight over time. Symbols indicate group means and error bars indicate standard error of the mean. Sample size = 8 male mice for all treatment groups.

To determine whether the increased production and release of FGF21 in response to cisplatin play any role in the wheel-running activity of mice, mice received daily injections of the KLB antagonist peptide 19 starting 1 day before cisplatin treatment and continuing for a total of 5 days (Figure 4). Mice ran an average of 35,689 ± 1,839 revolutions (mean ± SEM) per day at baseline. During the whole period of observation, from day 0 to day 14, cisplatin decreased wheel running activity (cisplatin factor F(1, 28) = 93.7, p < 0.001). Peptide 19 had no effect, both by itself (peptide 19: F(1, 28) = 0.94) and in interaction with cisplatin (cisplatin x peptide 19, F(1, 28) = 0.59). However, a closer inspection of the wheel running data shows that peptide 19 decreased voluntary wheel running activity during the days it was administered. This was confirmed by an ANOVA conducted on days 1–5, which showed a highly significant effect of peptide 19 (F(1, 28) = 11.0, p < 0.01) (Figure 4). There was no interaction between peptide 19 and cisplatin during this time (F(1, 28) = 2.40), indicating that both control mice and cisplatin-treated mice were affected. Recovery of wheel running after cisplatin treatment was unaffected by the peptide 19. Cisplatin induced a severe body weight loss (cisplatin F(1, 28) = 151, p < 0.001). Peptide 19 had no effect on its own (F(1, 28) = 0.94).

### 3.4 Peptide 19 potentiates the decrement in wheel running that develops in mEER tumor-bearing mice

**FIGURE 5:**
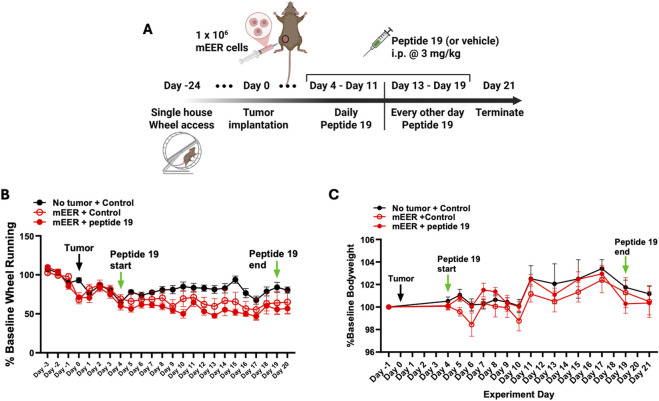
Peptide 19 in tumor-bearing mice. Schematic of experimental design and timeline for tumor implantation and Peptide 19 administration **(A)**. Line graph showing percent baseline wheel running over the course of tumor development and Peptide 19 treatment **(B)**. Line graph showing percent baseline bodyweight over the experimental timeline **(C)**. Sample sizes: No Tumor + Vehicle = 8 | mEER + Vehicle = 8 | mEER + Peptide 19 = 7.

To determine whether the increased production and release of FGF21 in response to cancer play a role in the decreased wheel-running activity observed in mEER tumor-bearing mice, they were treated daily with peptide 19, starting 4 days after implantation of mEER tumor cells (Figure 5). The baseline wheel running activity during the last 3 days before tumor implantation was 38,787 ± 1,838 revolutions (mean ± SEM). A two-way ANOVA (treatment x time) showed a significant effect of treatment (F(2, 20) = 3.92, p < 0.05) and time (F(15, 300) = 3.07, p < 0.001). There was no significant treatment x time interaction (F(30, 300) = 1.24). Post hoc comparison of the average wheel running across the three groups using the Fischer LSD test revealed that only the tumor-bearing mice treated with peptide 19 differed from the control mice that did not receive tumor cells (t(20) = 2.72, p < 0.05). The difference in wheel running activity between control mice and tumor-bearing mice not treated with peptide 19 was only trending (t(20) = 1.91, p = 0.07). These results suggest that peptide 19 exacerbated the decline in wheel running caused by tumor progression. Body weights did not differ among the three groups (F(2, 20) = 0.91). Peptide 19 had no impact on mEER tumor weight at the end of the experiment (mEER-control mice, n = 8, mean ± SEM 1.35 ± 0.15 g; mEER-peptide 19, n = 7, mean ± SEM 1.39 ± 0.13 g; t(13) = 0.21, NS).

## 4 Discussion

The results of the present experiments demonstrate that HPV-related head and neck cancer and the chemotherapeutic drug cisplatin elevate circulating FGF21 levels. Consistent with the liver being the primary source of circulating FGF21 (Trusz, 2024), mRNA expression of this mitokine was significantly increased in this organ in response to cisplatin. This was not observed in tumor-bearing mice. As cisplatin elevates both GDF15 and FGF21 and administration of exogenous GDF15 induces behavioral fatigue in mice (Chelette et al., 2023), we investigated whether FGF21 has a similar effect. This was clearly not the case, as administration of the FGF21 agonist LY2405319 did not reduce voluntary wheel-running activity. There are several possible explanations for the lack of effect of pharmacological activation of the FGF21 pathway, including pathway saturation, insufficient target engagement, or context-dependent responsiveness, all of which warrant further consideration. Whatever the case, this does not mean that FGF21 plays no role in wheel-running performance, as blocking its co-receptor with peptide 19 decreased this behavior in healthy mice. This finding indicates that, in already trained mice, activation of β-klotho is necessary for optimal performance. This observation is not surprising, given the physiological role of FGF21 in the metabolic adjustments associated with physical exercise (Abe and Dantzer, 2025). What remained unknown was whether FGF21 plays a similar role in situations where its levels are elevated due to cisplatin treatment or tumor growth, and voluntary wheel running is already reduced. The present findings show that inhibiting the activity of this mitokine on its cellular targets with peptide 19 worsened behavioral performance.

Although a substantial body of research exists on the role of FGF21 in metabolic adjustments during physical exercise (Abe and Dantzer, 2025), its role in facilitating physical activity remains uncertain. FGF21 expression in skeletal muscle during endurance exercise is essential for converting fast-twitch fibers into slow-twitch fibers in the gastrocnemius muscles (Luo et al., 2023). Mice with a genetic deletion of *Fgf21* exhibited a reduced ability to run on a treadmill at low speeds but not at high speeds, probably because of limited capacity to sustain effort over time (Luo et al., 2023). However, deleting *Fgf21* in mice did not affect their performance on a running wheel during the dark phase of the light-dark cycle, despite FGF21 having beneficial effects on muscle glucose metabolism and liver lipid metabolism (Loyd et al., 2016). Experiments involving constitutive deletion of *Fgf21* are difficult to interpret without a specific attempt to restore FGF21 to normal levels, as compensatory mechanisms involving other metabolically active factors are likely to occur.

The observation that peptide 19 exacerbates the effects of cancer and its treatment on voluntary wheel running does not necessarily mean that the role of FGF21 in these conditions is related to its ability to modulate the metabolic adjustments required by physical exercise. Our findings in mEER tumor-bearing mice, as well as in cisplatin-treated mice given peptide 19, could be explained by the ability of FGF21 to protect against cisplatin toxicity or to mitigate the metabolic consequences of tumor growth. There is already evidence that the increased production of FGF21 in response to cisplatin acts as a protective mechanism against kidney and liver toxicity caused by this chemotherapeutic agent (Li et al., 2018; Chen et al., 2020; Zhang et al., 2021). As cisplatin-induced cellular stress leads to mitochondrial dysfunction (Oliveira et al., 2024), this is likely another example of the ability of FGF21 to regulate the mitochondrial integrated stress response in an autocrine and endocrine manner (Forsstrom et al., 2019). Circulating levels of FGF21 are consistently elevated in primary mitochondrial disorders, making this mitokine a promising biomarker for diagnosing this disease category (Lin et al., 2020). The role of FGF21 in tumor progression is complex and varies with disease type and stage (Dai et al., 2021; Lu et al., 2021; Sui et al., 2024). In some cancers, it promotes tumor growth, whereas in others it suppresses cell proliferation and promotes apoptosis. Serum levels of FGF21 are elevated in breast cancer, renal cancer, and endometrial cancer, although its role, if any, in the development of these cancers remains unknown (Phan et al., 2021). Fibroblast growth factors have been associated with HPV-related cervical cancer (Mahmood et al., 2022). However, this effect involves the non-endocrine family of fibroblast growth factors that does not act through the β-klotho co-receptor. Although we did not systematically explore the origin of the increased FGF21 levels observed in mEER tumor-bearing mice, it is still possible that elevated expression of FGF21 in the tumor itself is responsible.

Despite the evidence provided by the present findings for a role of β-klotho in counteracting the impaired energy metabolism caused by mEER tumor growth and cisplatin, this study has some limitations in its conclusions. The first is the lack of consideration for potential sex differences, as experiments were conducted only in male mice. This is because the mEER tumor cell line we used was initially developed from epithelial oropharyngeal cells of a male mouse and is therefore rejected by female mice. We chose to use the mEER tumor cell line despite this limitation because the tumor is characterized by a very high glycolytic metabolism, creating an ideal condition to observe the potential relevance of metabolically active factors like FGF21 (Coppock et al., 2013). Another limitation is the focus on β-klotho. As its expression as a co-receptor of FGF21 varies with the organism’s metabolic status (Abe and Dantzer, 2025), it would have been necessary to examine its expression at the protein level in the metabolic organs targeted by FGF21 to fully interpret the present data. In addition, blockade of β-klotho with peptide 19 could interfere with other endocrine fibroblast growth factors such as FGF19 and FGF23 that use the same co-receptor (Phan et al., 2021). Because FGF23 mainly regulates phosphate homeostasis, it is unlikely to be involved in the metabolic changes and mitochondrial dysfunction underlying behavioral fatigue observed in this study. However, this is not the case for FGF19. Although FGF19 primarily functions during embryogenesis, it is also present in adulthood. Its physiological expression is limited to the ileum, from where it acts on the liver to regulate bile acid synthesis. FGF19 has been proposed as a driver of certain cancers, such as hepatocellular carcinoma and lung squamous cell carcinoma (Tan et al., 2016; Wu et al., 2011). Additionally, it plays a protective role in skeletal muscle fibers and mitochondria, although this effect has been primarily studied in the context of obesity (Guo et al., 2020).

Overall, this study shows that the adverse effects of peptide 19 on behavioral fatigue in mEER tumor-bearing mice and cisplatin-treated mice highlight a protective role for β-klotho activation by endocrine fibroblast growth factors in these conditions. We suggest that this activation helps counteract the metabolic alterations caused by tumor growth and the mitochondrial damage induced by chemotherapeutic agents such as cisplatin. The specific cellular and molecular mechanisms underlying the effects of fibroblast growth factors in these conditions remain to be investigated.

## Data Availability

The raw data supporting the conclusions of this article will be made available by the authors, without undue reservation.
