# Supplementary material for: Antagonism of β-klotho signaling by peptide 19 impairs wheel running in male mice and potentiates the cisplatin-induced decrease in wheel running
Source: Front Pharmacol. 2026 Jun 26;17:1800474. doi: 10.3389/fphar.2026.1800474 (PMC13350045; doi:10.3389/fphar.2026.1800474)
Supplement: Supplementary file 1 [file Supplementaryfile1.docx]

**Supplementary Material**

**Supplementary Table I**

**Detailed statistical analysis**

**Experiment I: Effects of cisplatin on FGF21 plasma levels and Fgf21 mRNA expression in the liver (Student’s t-test)**

FGF21 plasma levels t(9)=3.3 p<0.01

Fgf21 mRNA liver t(10)=4.2 p<0.01

**Experiment II : Effect of mEER tumor on FGF21 plasma levels and Fgf21 mRNA expression in the liver (Student t-test)**

FGF21 plasma levels t(14)=5.9 p<0.001

Fgf21 mRNA liver t(14)=0.71 NS

**Experiment III : Effects of two doses of LY2405319 on daily wheel running and body weight of healthy mice, expressed as a percentage of baseline (three-way ANOVA: LY2405319 x peptide 19 x time)**

Wheel running, 3 mg/kg LY2405319

LY2405319 F(1, 19)=074 NS

Peptide 19 F(1, 19)=0.25 NS

LY2405319 x Peptide 19 F(1, 19)=1.16 NS

Time F(14, 266)=18.5 p<0.001

LY2405319 x time F(14, 266)=0.71 NS

Peptide 19 x time F(14, 266)=0.34 NS

LY2405319 x Peptide 19 x time F(14, 266)=1.53 NS

Wheel running,7.5 mg/kg LY2405319

LY2405319 F(1, 20)=052 NS

Peptide 19 F(1, 20)=1.24 NS

LY2405319 x Peptide 19 F(1, 20)=0.01 NS

Time F(9, 180)=3.57 p<0.001

LY2405319 x time F(9, 180)=1.23 NS

Peptide 19 x time F(9, 180)=0.34 NS

LY2405319 x Peptide 19 x time F(9, 180)=0.55 NS

Body weight, 3 mg/kg LY2405319

LY2405319 F(1, 20)=0.10 NS

Peptide 19 F(1, 20)=2.82 NS

LY2405319 x Peptide 19 F(1, 20)=0.73 NS

Time F(5, 100)=2.59 p<0.001

LY2405319 x time F(5, 100)=0.08 NS

Peptide 19 x time F(5, 100)=1.53 NS

LY2405319 x Peptide 19 x time F(5, 100)=1.01 NS

**Body weight, 7.5 mg/kg LY2405319**

LY2405319 F(1, 20)=2.10 NS

Peptide 19 F(1, 20)=0.20 NS

LY2405319 x Peptide 19 F(1, 20)=0.63 NS

Time F(4, 80)=12.2 p<0.001

LY2405319 x time F(4, 80)=3.77 p<0.01

Peptide 19 x time F(4, 80)=0.44 NS

LY2405319 x Peptide 19 x time F(4, 80)=1.01 NS

**Experiment IV: Effects of peptide 19 on cisplatin-induced changes in wheel running and body weight expressed as a percentage of baseline**

**Wheel running (days 0 to 5)**

Peptide 19 F(1, 28)=1.23 NS

Cisplatin F(1, 28)=93.7 p<0.001

Peptide 19 x cisplatin F(1, 28)=0.59 NS

Time F(17,476)=36.8 p<0.001

Peptide 19 x time F(17, 476)=2.21 p<0.05

Cisplatin x time F(17, 476)=41.8 p<0.001

Peptide 19 x cisplatin x time F(17, 476)= 1.03 NS

**Body weight (days -1 to 14)**

Peptide 19 F(1, 28)=0.94 NS

Cisplatin F(1, 28)=151 p<0.001

Peptide 19 x cisplatin F(1, 28)=4.86 p<0.05

Time F(15, 420)=79.3 p<0.001

Peptide 19 x time F(15, 420)=0.95 NS

Cisplatin x time F(15, 420)=73.4 p<0.001

Peptide 19 x cisplatin x time F(15, 420)=1.18 NS

**Experiment V: Effect of peptide 19 on tumor-induced changes in daily wheel running (days 5 to 20) and body weights expressed as a percentage of baseline (2-way ANOVA (treatment x time)**

**Wheel running**

Treatment F(2, 20)=3.92 <0.05

Time F(15, 300)=3.07 <0.001

Treatment x day F(30, 300)=1.24 NS

Post-hoc comparisons for treatment effects (Fischer LSD test):

No tumor/control vs. mEER/control: t(20)=1.91 p=0.07

No tumor/control vs. mEER/peptide 19: t(20)=2.72 p<0.05

mEER/control vs. mEER/peptide 19: t(20)=0.87 NS

**Body weight**

Treatment F(2, 20)=0.91 NS

Time F(13, 260)=6.25 p<0.001

Treatment x time F(26, 260)=0.49 NS
